# A Time Series Analysis: Weather Factors, Human Migration and Malaria Cases in Endemic Area of Purworejo, Indonesia, 2005–2014

**Published:** 2018-04

**Authors:** Dwi Sarwani Sri REJEKI, Nunung NURHAYATI, Budi AJI, E. Elsa Herdiana MURHANDARWATI, Hari KUSNANTO

**Affiliations:** 1. Dept. of Public Health, Faculty of Health Sciences, Universitas Jenderal Soedirman, Purwokerto, Indonesia; 2. Dept. of Mathematics, Faculty of Mathematics and Science, Universitas Jenderal Soedirman, Purwokerto, Indonesia; 3. Center for Tropical Medicine and Department of Parasitology, Faculty of Medicine, Universitas Gadjah Mada, Yogyakarta, Indonesia; 4. Dept. of Public Health, Faculty of Medicine, Universitas Gadjah Mada, Yogyakarta, Indonesia

**Keywords:** Malaria, Weather factors, Human migration, Climate change, Time series analysis, Indonesia

## Abstract

**Background:**

Climatic and weather factors become important determinants of vector-borne diseases transmission like malaria. This study aimed to prove relationships between weather factors with considering human migration and previous case findings and malaria cases in endemic areas in Purworejo during 2005–2014.

**Methods:**

This study employed ecological time series analysis by using monthly data. The independent variables were the maximum temperature, minimum temperature, maximum humidity, minimum humidity, precipitation, human migration, and previous malaria cases, while the dependent variable was positive malaria cases. Three models of count data regression analysis i.e. Poisson model, quasi-Poisson model, and negative binomial model were applied to measure the relationship. The least Akaike Information Criteria (AIC) value was also performed to find the best model. Negative binomial regression analysis was considered as the best model.

**Results:**

The model showed that humidity (lag 2), precipitation (lag 3), precipitation (lag 12), migration (lag1) and previous malaria cases (lag 12) had a significant relationship with malaria cases.

**Conclusion:**

Weather, migration and previous malaria cases factors need to be considered as prominent indicators for the increase of malaria case projection.

## Introduction

Global climate change has serious implications for the spread of infectious diseases, including vector-borne diseases such as malaria and dengue (1, 2). Climate change directly affects the vector-borne disease’s transmission by altering the geographical range of vector, increasing numbers of reproduction and biting rate, and also shortening pathogenic incubation period of parasite (3). Climatic and weather factors become important determinants of malaria transmission. Changes in climatic and weather variables including temperature, precipitation, and relative humidity influence the population of *Anopheles* mosquitoes and malaria occurrence (4, 5). Warmer ambient temperatures affect the extrinsic cycle by shortening its duration, so enhancing the transmission potential of malaria (6, 7). Temperature was associated with malaria cases in Korea, Southwestern China, India, West Africa, Jimma of South West Ethiopia and South Ethiopia (8–13). A 1°C increase in temperature was associated with 18% increase in malaria cases after a 3-wk lag (8). Conversely, temperatures in West Africa and Zimbabwe had a significant negative correlation with the incidence of malaria (11, 14), while in the highlands of Bangladesh, the temperature had no effect on malaria cases (15).

Precipitation plays a major role in the transmission of malaria although it has no direct effect on parasite. Rain water creates pools that can become a breeding site for *Anopheles* mosquitoes. High rainfall also increases humidity and prolongs the life of adult mosquitoes. In Sub-Saharan Africa, malaria has a higher transmission rate during the rainy season (11). The increases of precipitation and humidity are directly proportional to the density of *Anopheles* mosquitoes (16). In India, higher malaria cases were reported when precipitation was high (9), while in Tibet, precipitation was an important factor in the transmission of malaria. Furthermore, precipitation and incidence of malaria have a complex and non-linear relationship (5). The morbidity and mortality of patients with malaria were significantly increased after a flood event in Pakistan (17). In Tibet, Jimma of South West Ethiopia, Ghana and Shucher of China showed that rain was associated with malaria (5, 12, 18, 19). Otherwise, in Bangladesh, precipitation was not associated with the malaria cases (15).

A direct effect of humidity on the life cycle and biting behavior of mosquitoes was reported (9). In Indonesia, humidity had a significant association with the density of *Anopheles* (16). Low relative humidity shortens the lifespan of mosquitoes; otherwise high relative humidity extends the lifespan of the mosquitoes. At higher humidity, the mosquitoes become more active and more likely to bite. In Tibet, India, Ethiopia, and China showed humidity affected the incidence of malaria (5, 10, 12, 19). Conversely, in Bangladesh, humidity had no effect on malaria cases (15).

Human migration has contributed to the spread of malaria transmission (20–23). Failure to consider this factor may contribute to failure of predicting the increase malaria case. A long-term migration data can be used to predict the change of the people who are infected by malaria (24). In Abidjan and Burkina Faso, human population movement to endemic areas leads to the increase of malaria case (25, 26). However, a contrary finding was confirmed human migration in developing countries decreased malaria cases (22).

In Indonesia, malaria remains a public health problem, although the incidence of malaria was decreased by 1.9% in 2013 compared with 2.9% in 2007. However, some provinces still have a high annual parasite incidence (API) that are potential for the higher risk of transmission and outbreaks (27). As a disease highly sensitive to climate change, malaria in Indonesia is predicted to have a significant effect when there are changes in climatic and weather variables particularly in the endemic areas (28). Therefore, the present study aimed to analyze the relationship between weather factors with considering human migration and previous case findings and malaria cases in endemic areas in Indonesia.

This study was focusing on the endemic areas in the district of Purworejo, a highest malaria-endemic district in Central Java Province, with an API of 1.34 per 1000 population in 2011 (29).

## Methods

### Study site

**Fig. 1:**
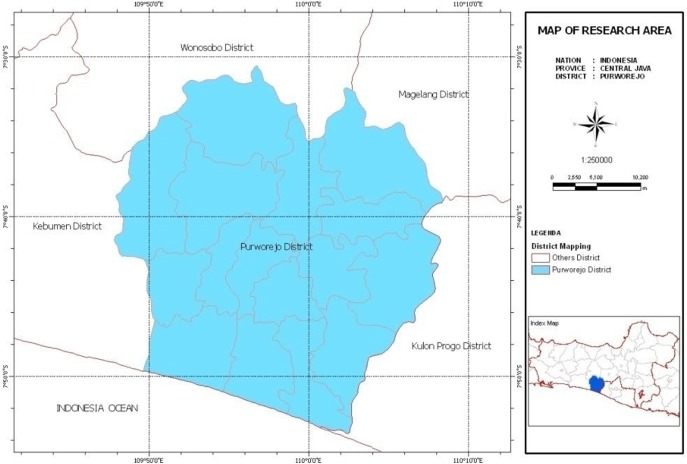
Location of the district of Purworejo, Central Java, Indonesia

Purworejo is one of the districts in Central Java Province, Indonesia and geographically is located in between 109°47′28″ longitude and 110°8′20″East longitude (EL), and 7°32′ latitude and 7°54′ South latitude (SL) (Fig. 1). Purworejo consists of 16 sub-districts and 494 villages, with a total area of 1034.82 km^2^. Total population was 899126 people in 2012 with 0.24 percent/year of population growth and 868.40 inhabitants/km^2^ of population density 23. Climate variables in Purworejo, like other regions in Indonesia, are influenced by three factors: the change in the position of the sun during revolution, northeast monsoon, and the southeast monsoon winds. Both of monsoon winds are the most dominating factors of the Indonesian climate. Southeast monsoon winds blow from Australia and bring dry weather and drought in Purworejo region, while Northeast monsoon winds blow from Asia to Australia and bring rain. During the period of 1996 to 2012, there were 4 dry season months in Purworejo with less than 60 mm rainfall from Jun to Sep; and 8 rainy season months with more than 100 mm precipitation (30).

### Study design and data analysis

The study was based on an ecological study with cross-sectional approach by using monthly time series data from 10 yr period. The independent variables were the maximum and minimum temperatures, maximum and minimum humidity, precipitation, human migration and previous malaria cases, while the dependent variable was positive malaria cases. The study population was all positive malaria cases reported in the district health office of Purworejo. The samples were all positive malaria cases reported and recorded in the district health office of Purworejo from 2005 to 2014. Maximum and minimum temperatures, maximum and minimum humidity, and precipitation data were collected from PROBOLO Central Management of Water Resources, Central Java Province. Human migration data were collected from the civil registry office of Purworejo, while data on the number of positive malaria cases collected from the monthly report of malaria surveillance of the district health office of Purworejo. All independent and dependent variables data were collected for the 10 yr from 2005 to 2014. Three models of count data regression analysis i.e. Poisson model, quasi-Poisson model, and negative binomial model were applied to measure the relationship. To find the best model, the least AIC value was also performed. This analysis was facilitated using statistical software R 3.2.2.

Ethical approval was received from the Ethical Committee of the Faculty of Medicine, Gadjah Mada University, Indonesia. Permission to carry out the study was also obtained from Purworejo District Government.

## Results

**Fig. 2:**
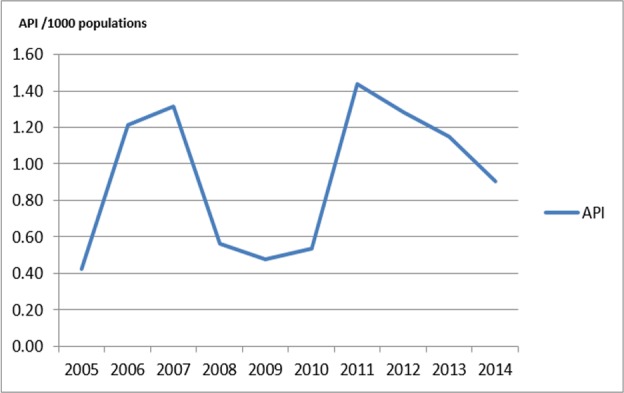
API Trend in the district of Purworejo, Indonesia from 2005 to 2014

Malaria cases in Purworejo tended to decline over the last 10 yr. However, malaria is still a major public health problem in Purworejo, and outbreaks sometimes occur in this area 23. Fig. 2 shows the number of malaria cases in Purworejo from 2005 to 2014. API was fluctuated within the last 10 yr, with an API >1/1000 in 2006–2007 and 2011–2013, and there were years with cases amounted to nearly 1000 patients. Most malaria cases were in the sub-districts of Kaligesing, Bagelen, and Bener located on the border of the districts of Kulonprogo and Purworejo.

Mean yearly minimum temperatures of 2005–2014 range from 18.8 °C to 27.2 °C, with the highest minimum temperature, were reached in 2009 (Table 1). Mean yearly maximum temperature range from 28.3 °C to 29.9 °C, with the highest maximum temperature was in 2010, and the lowest was in 2007. Mean minimum humidity in 2005–2014 range from 72.3% to 83.5% with the lowest minimum humidity occurred in 2011 and the highest in 2005. Mean maximum humidity in 2005–2014 range from 91.3% to 96.3%, while the average rainfalls range from 177.7 to 251.3 mm.

**Table 1:** Characteristics of weather in Purworejo from 2005 to 2014

	***2005***	***2006***	***2007***	***2008***	***2009***	***2010***	***2011***	***2012***	***2013***	***2014***
Min T										
- Mean	19.8	23.4	23.2	22.4	27.2	21.0	18.8	20.6	20.4	20.2
- Min	17.4	21.1	21.0	20.2	19.4	19.4	16.6	18.9	19.1	18.1
- Max	22.8	25.2	24.6	23.4	22.6	22.2	21.3	22.4	20.9	21.1
Max T										
- Mean	29.6	28.6	28.3	28.6	29.2	29.9	28.9	29.1	29.2	29.7
- Min	28.9	27.0	24.2	26.8	27.5	29.2	27.2	28.2	27.8	28.6
- Max	30.8	30.3	29.9	29.6	30.3	30.7	29.9	30.2	30.3	30.7
Minimum humidity										
- Mean	83.5	81.1	80.8	80.6	75.1	77.8	72.3	75.2	73.1	73.8
- Min	81.0	74.0	74.0	77.0	69.0	70.0	69.0	68.0	32.0	68.0
- Max	85.0	87.0	87.0	85.0	80.0	83.0	77.0	81.0	83.0	81.0
Maximum humidity										
- Mean	93.4	95.5	96.3	96.1	95.2	95.8	91.3	94.7	94.9	92.4
- Min	92.0	94.0	93.0	93.0	91.0	91.0	79.0	92.0	89.0	83.0
- Max	97.0	99.0	99.0	99.0	99.0	98.0	97.0	99.0	99.0	99.0
Precipitation										
- Mean	225.3	194.8	233.4	220.0	177.7	327.4	238.4	211.0	251.3	189.8
- Min	11.0	0	0	0	0	81.0	0	0	0	0
- Max	683.0	458.0	920.0	462.0	490.0	500.0	544.0	530.0	625.0	627.0

**Fig. 3:**
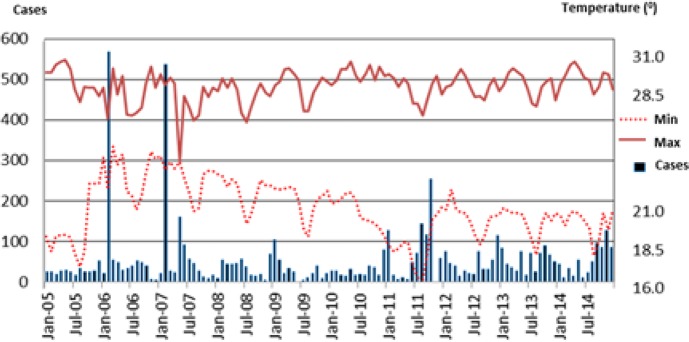
Malaria cases, minimum, and maximum temperatures in the district of Purworejo, Indonesia from 2005 to 2014

Fig. 3 shows the trend of malaria cases, maximum, and minimum temperatures in Purworejo during 2005–2014. Malaria cases were exhibited a fluctuating trend. The cases increased during Nov–Apr and decreased during May–Sep.

The highest number of cases occurred in Feb 2006 and Feb 2007. In 2011, malaria cases tended to increase in Aug–Oct, and the highest number of cases occurred in Nov 2014. The mean minimum and maximum temperatures for every month were nearly the same, where, the mean monthly minimum temperature for 10 yr was 21.1 °C and the mean maximum temperature was 29.1 °C.

**Fig. 4:**
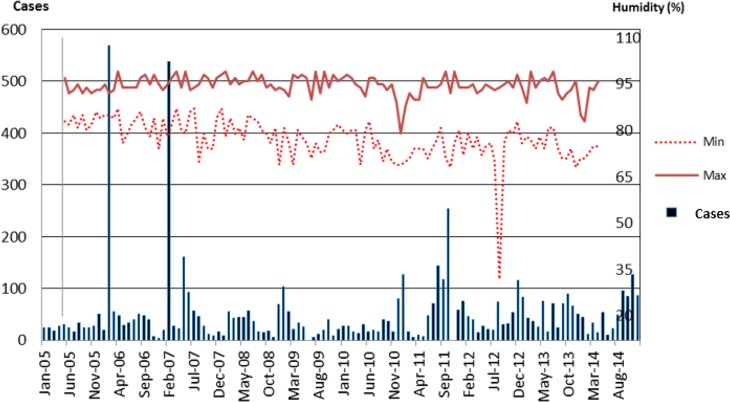
Malaria cases along with minimum and maximum humidity in the district of Purworejo, Indonesia from 2005 to 2014

Figure 4 shows the trend of malaria cases along with the minimum and maximum temperatures and humidity in Purworejo from 2005 to 2014. The mean monthly minimum humidity for 10 yr was 77.3% and the mean maximum humidity was 94.6%. In general, the minimum and maximum humidity pattern were almost the same, but there was little difference in Feb 2013 where the minimum humidity was quite low at 32%. The highest maximum humidity was in Feb and Apr 2007, while the lowest minimum humidity was in Feb 2013.

**Fig. 5:**
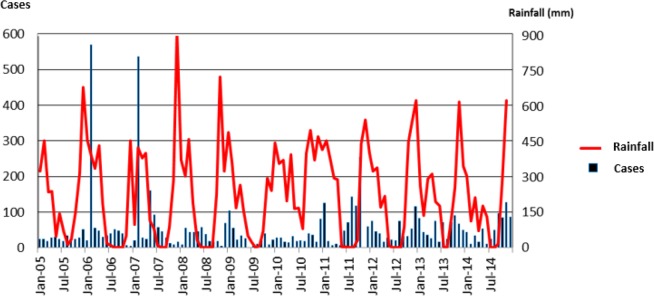
Malaria cases and precipitation in the district of Purworejo, Indonesia from 2005 to 2014

Figure 5 depicts the trend of malaria cases and precipitation during 2005–2014. The malaria cases and precipitation patterns were almost similar. Malaria cases and precipitation were more likely to increase in Jan and decrease in July. The mean monthly precipitation for 10 yr was 226.9 mm, with the most precipitation amount occurred in Dec 2007 was 920 mm and the lowest in Aug–Sep for whole year.

**Fig. 6:**
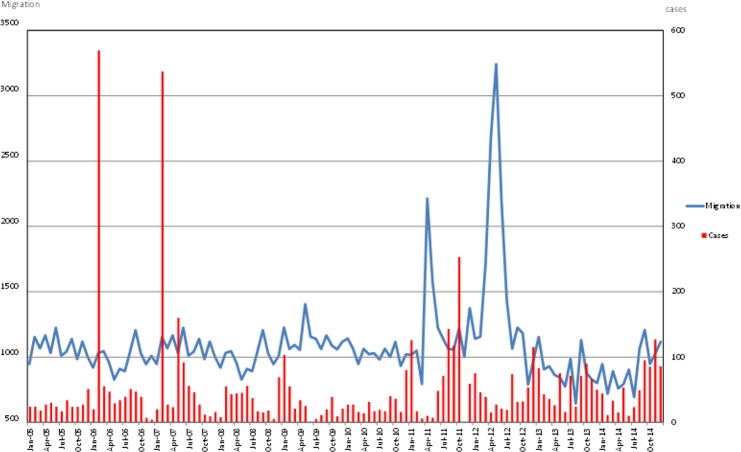
Malaria cases and human migration in the district of Purworejo, Indonesia from 2005 to 2014

Figure 6 depicts the trend of malaria cases and human migration during 2005–2014. Malaria cases tended to decrease while the inward migration increased. Although there was not a clear pattern of the human migration the decreased inward migration occurred during Jun to Aug, and the highest inward migration was in May 2012.

Table 2 reports the results of all three multivariate regression models (Poisson regression, quasi-Poisson and negative binomial.

**Table 2:** Analysis of weather factors and malaria cases using three models: Poisson regression, quasi-Poisson, and negative binomial

		**Model 1**	**Model 2**	**Model 3**
	(Intercept)	3.391e+01	3.391e+01	27.4230513
		< 2e-16 ^***^	0.000574 ^***^	0.000134 ^***^
1	T_min	8.245e-02	8.245e-02	0.0449369
		0.000907 ^***^	0.515172	0.688686
2	T_min_lag1	9.581e-02	9.581e-02	−0.0094624
		0.000305 ^***^	0.478902	0.937848
3	T_min_lag2	−3.090e-01	−3.090e-01	−0.2099637
		< 2e-16 ^***^	0.038480 ^*^	0.092559
4	T_min_lag3	7.486e-02	7.486e-02	0.0883774
		0.004932 ^**^	0.581148	0.413756
5	T_min_lag12	−7.650e-02	−7.650e-02	−0.0745271
		3.11e-13 ^***^	0.155007	0.094101
6	T_max	−2.934e-01	−2.934e-01	−0.1920551
		< 2e-16 ^***^	0.019327 ^*^	0.061779
7	T_max_lag1	−1.180e-01	−1.180e-01	−0.1318423
		6.21e-07 ^***^	0.329070	0.190029
8	T_max_lag2	−6.735e-02	−6.735e-02	−0.0294904
		0.007689 ^**^	0.600893	0.771033
9	T_max_lag3	1.738e-01	1.738e-01	0.0896790
		1.66e-11 ^***^	0.188593	0.375389
10	T_max_lag12	−1.270e-01	−1.270e-01	−0.0588534
		1.05e-10 ^***^	0.206919	0.509201
11	Humidity_max	−2.973e-02	−2.973e-02	−0.0436958
		7.12e-06 ^***^	0.379017	0.083258
12	Humidity_max_lag1	2.590e-03	2.590e-03	−0.0006585
		0.645719	0.928044	0.977646
13	Humidity_max_lag2	−1.154e-01	−1.154e-01	−0.1040783
		< 2e-16 ^***^	0.000761 ^***^	1.98e-05 ^***^
14	Humidity_max_lag3	−4.391e-02	−4.391e-02	−0.0392618
		6.31e-16 ^***^	0.115405	0.109481
15	Humidity_max_lag12	−3.151e-02	−3.151e-02	0.0018897
		1.27e-05 ^***^	0.392383	0.945133
16	Humidity_min	−1.051e-02	−1.051e-02	−0.0068449
		0.000599 ^***^	0.500874	0.578533
17	Humidity_min_lag1	2.435e-02	2.435e-02	0.0221173
		2.10e-11 ^***^	0.190810	0.075625
18	Humidity_min_lag2	1.847e-02	1.847e-02	0.0238382
		4.01e-07 ^***^	0.321064	0.078312
19	Humidity_min_lag3	2.263e-02	2.263e-02	0.0241512
		1.60e-10 ^***^	0.211440	0.087376
20	Humidity_min_lag12	3.411e-03	3.411e-03	0.0051224
		0.289942	0.835341	0.669126
21	Precipitation	8.711e-04	8.711e-04	0.0007136
		2.46e-11 ^***^	0.192322	0.173781
22	Precipitation_lag1	−9.428e-04	−9.428e-04	−0.0005943
		9.80e-11 ^***^	0.206228	0.289205
23	Precipitation_lag2	4.726e-04	4.726e-04	0.0001873
		0.000114 ^***^	0.449448	0.746189
24	Precipitation_lag3	8.645e-04	8.645e-04	0.0010392
		1.18e-11 ^***^	0.185371	0.041674 ^*^
25	Precipitation_lag12	2.102e-03	2.102e-03	0.0017799
		< 2e-16 ^***^	0.001815 ^**^	0.000416 ^***^
26	Cases_lag1	1.564e-03	1.564e-03	0.0007621
		2.87e-10 ^***^	0.217856	0.453601
27	Cases_lag2	−9.954e-04	−9.954e-04	−0.0005112
		1.93e-05 ^***^	0.402443	0.585365
28	Cases_lag3	1.614e-03	1.614e-03	0.0015659
		3.94e-09 ^***^	0.249666	0.162524
29	Cases_lag_12	3.195e-03	3.195e-03	0.0037091
		< 2e-16 ^***^	0.000302 ^***^	5.66e-05 ^***^
30	Migration	1.851e-04	1.851e-04	0.0002141
		0.009005 ^**^	0.608215	0.449025
31	Migration_lag1	−7.150e-04	−7.150e-04	−0.0006733
		1.34e-15 ^***^	0.119540	0.044296 ^*^
32	Migration_lag2	−5.227e-05	−5.227e-05	0.0003584
		0.527216	0.901159	0.298063
33	Migration_lag3	−4.632e-05	−4.632e-05	−0.0002814
		0.438840	0.879135	0.304673
34	Migration_lag12	7.903e-04	7.903e-04	0.0007283
		< 2e-16 ^***^	0.027612 ^*^	0.007040 ^**^
	AIC	2406.7	NA	1047.9

Note.

* <0.05,

** <0.01,

*** <0.001 significant levels.

Poisson regression model (Model 1) produced more significant predictor variables; however, Model 1 was not appropriate enough to be applied due to its inability to detect a potential problem with overdispersion. Then, to identify the problem of overdispersion, quasi-Poisson regression (Model 2) was also conducted. The estimated dispersion parameter for quasi-Poisson was more than 1, indicating over-dispersion; therefore, negative binomial regression (Model 3) was selected. Further, the least AIC value of negative binomial regression was smaller than those from quasi-Poisson. Based on negative binomial regression analysis, there were significant relationships between four predictor variables (i.e, maximum temperature, maximum humidity in the last 2 months, precipitation in the last 3 and 12 months, previous malaria cases findings in the last 12 months and migration in the last 1 month) with malaria cases. Table 3 shows the results of negative binomial regression analysis as the best model to determine the relationship between weather factors with considering previous case findings and malaria cases in Purworejo from 2005 to 2014 by using monthly time series data.

**Table 3:** The best models of weather factors and malaria cases using negative binomial regression

		***Estimate***	***Std. Error***	***Z value***	***Pr(>|z|)***
	Intercept	14.1954025	2.2651468	6.267	3.68e-10 ^***^
	Humidity max lag2	−0.1106626	0.0235174	−4.706	2.53e-06 ^***^
	Precipitation lag 3	0.0008549	0.0003972	2.152	0.031381 ^*^
	Precipitation lag 12	0.0009424	0.0003950	2.386	0.017033 ^*^
	Cases lag 12	0.0035568	0.0009946	3.576	0.000349 ^***^
	Migration	−0.0004730	0.0002349	−2.014	0.044043 ^*^

Note.

* <0.05,

** <0.01,

*** <0.001 significant levels

## Discussion

The present study showed significant relationships between weather factors with considering human migration and previous case findings and malaria cases in endemic areas in Purworejo, exclusively by using negative binomial regression model. Maximum humidity lag 2 had significant relationship with malaria cases, and the relationship was negative. Maximum humidity in the last 2 months reduced positive malaria cases. A 1% increase in maximum humidity was associated with a 10.47% decrease in malaria cases after a 2-months lag. This result is consistent with previous studies in Korea that relative humidity associated with 40.7% decrease in malaria after a 7-wk lag (8). Humidity influences the life cycle of the mosquito, human-biting habit, and the density of the mosquito. Low relative humidity shortens the life of the mosquito, while high relative humidity prolongs its life. Higher humidity increase the number of times mosquito bite, and the density of mosquitoes (31). Relative humidity of at least 60% needed for survival of adult *Anopheles* and transmitting the parasite (10).

This study also explains the relationship between precipitation lag 3, precipitation lag 12 and malaria cases. A 1 mm increase in precipitation was associated with a 0.08% increase in malaria cases after a 3-months lag. A 1 mm increase in precipitation was associated with a 0.09% increase in malaria cases after a 12-months lag. This study result is in line with study findings conducted in Jimma of Ethiopia, Ghana and China that reported significant effects of the level of precipitation on malaria (12, 18, 19). Precipitation is a prominent factor in the transmission of malaria. Rainfall influences the transmission by supplying water to create aquatic habitat, but excessive rainfall may wash off the breeding places and eliminate the larvae (16). Moreover, high intensity of rainfall causes the increase of the relative humidity that also influences malaria transmission by its role in prolonging mosquito life-span (12, 16). The present study provides evidence that human migration had significant relationship with malaria cases, and the relationship was negative. Inward migration reduced malaria cases. A 1% increase in inward migration was associated with a 0.047% decrease in malaria cases. Inward migration in Purworejo was accompanied with adequate sanitation that might lead to a decrease in malaria cases through reduction vector breeding sites. People who came to Purworejo were mostly in rainy season for preparing the land for planting. This activity could reduce the breeding sites and have also impact on deceasing malaria cases. This finding is line with previous study that human migration in developing countries supported by sufficient housing and sanitation would decrease malaria cases. Reduction of the human-vector contact and vector breeding sites were the plausible reasons for this phenomenon (22).

The study reveals highest relationship between previous malaria cases findings in the last 12 months with the current malaria cases. A 1 case increase in malaria cases was associated with a 0.36% increase in malaria cases after a 12-months lag. Malaria transmission was seasonal and influenced by the prior cases. Malaria incidence occurred in certain months and seasons when the density of Anopheles mosquitos in the previous period was much higher (10, 16). *An. balabacensis* malaria transmission in Sebatik island in Indonesia was quite high from Jun to Oct (32). Further, in Zimbabwe, malaria transmission started from Feb to May with the peak in Apr and lowest from Jul to Dec. This transmission pattern was closely related to geographic variation in climatic seasonality indicators, i.e. precipitation and temperature (33).

An important limitation of this study is related to public health interventions. This study did not incorporate public health interventions such as long-lasting insecticide-treated nets and indoor residual spraying in the models due to unavailability of the data and might have influenced the results of analysis.

## Conclusion

Weather factors such as temperature, humidity, precipitation, and considering previous malaria case findings need to be considered as prominent indicators for the increase of malaria case projection in Purworejo. This finding can conceivably be extended to other areas in Indonesia and elsewhere. However, the complex correlation among these variables should be elaborated with detailed ecological and epidemiological studies by taking into account local specific characteristics of the endemic areas.

## Ethical considerations

Ethical issues (Including plagiarism, informed consent, misconduct, data fabrication and/or falsification, double publication and/or submission, redundancy, etc.) have been completely observed by the authors.
